# Comparative transcriptomic and genomic analysis of tumor cells in the marginal and center regions of tumor nests in human hepatocellular carcinoma

**DOI:** 10.3389/fcell.2025.1611951

**Published:** 2025-07-16

**Authors:** Ziyi Li, Yikai Hu, Zhuotian He, Heyi Xu, Hongyang Wang, Yufei He

**Affiliations:** ^1^ Institute of Metabolism and Integrative Biology, Fudan University, Shanghai, China; ^2^ National Center for Liver Cancer and International Cooperation Laboratory on Signal Transduction, Eastern Hepatobiliary Surgery Institute/Hospital, Shanghai, China; ^3^ Fudan University Shanghai Cancer Center, Department of Oncology, Shanghai Medical College, Fudan University, Shanghai, China; ^4^ High School Affiliated to Shanghai Jiao Tong University, Jiading Campus, Shanghai, China; ^5^ Ulink College of Shanghai, Shanghai, China; ^6^ Molecular Pathology Laboratory, National Center for Liver Cancer, Eastern Hepatobiliary Surgery Hospital, Shanghai, China

**Keywords:** hepatocellular carcinoma, tumor nest, spatial transcriptomic, fibrotic nodules, heterogeneity

## Abstract

**Background:**

The tumor nests in solid tumors, including hepatocellular carcinoma (HCC), possess tumor-initiating potential, with the capacity to metastasize and form new metastatic lesions. However, the biological characteristics and heterogeneity of tumor cells at the central and marginal regions of these tumor nests remain poorly understood.

**Method:**

Based on pathological tissue sections, data integration and dimensionality reduction, we defined the boundaries and centers of tumor nests and fibrous nodules within 19 spatial transcriptomics (ST) samples from 8 HCC patients. Differential gene expression was analyzed at the single-unit, sample, patient, and pseudobulk levels, followed by Gene Ontology (GO) enrichment analysis. Additionally, spatial copy number variation (CNV) was inferred using inferCNV, and comparisons were made at the single-unit, sample, patient, and pseudobulk levels.

**Results:**

Ultimately, 24 tumor nests and 15 liver fibrosis nodules were analyzed. The spatial gene expression patterns of the tumor nests exhibited significant heterogeneity, with gene enrichment analysis revealing upregulation of immune-related pathways (e.g., humoral immune response mediated by circulating immunoglobulin; B cell receptor signaling pathway, etc.) at the tumor nest margins and growth/metabolism-related pathways (e.g., sulfur amino acid metabolic process; proteinogenic amino acid metabolic process, etc.) within the tumor nest center. Similar expression patterns were also observed in liver fibrous nodule samples. CNV and clustering analyses demonstrated transcriptional differences between tumor nests within individual patients, suggesting the evolutionary diversity, or heterogeneity, of tumor nests within the same tumor.

**Conclusion:**

Tumor nests exhibit significant transcriptional differentiation along spatial axes: the central regions show high expression of metabolism-related genes, while the marginal regions are enriched in immune-regulatory genes. This pattern is also observed in liver fibrotic nodules. This center-margin functional division may inform rational design of therapeutics that simultaneously modulate metabolism and immune responses.

## Introduction

HCC is a major global health concern, ranking sixth in incidence and third in cancer-related mortality worldwide. It is projected that between 2020 and 2040, the annual number of new liver cancer cases will increase by 55%, reaching 1.4 million diagnoses by 2040. Similarly, liver cancer-related deaths are expected to rise by 56%, with an estimated 1.3 million deaths in 2040 (Abboud et al., 2024; Rumgay et al., 2022). Over 90% of HCC cases are caused by chronic inflammatory liver diseases, with cirrhosis being the major risk factor. Among individuals with cirrhosis, the annual incidence of HCC ranges from 1% to 6%, making it the leading cause of death in this population (Marrero et al., 2018). HCC treatment options are diverse, including surgical resection, liver transplantation, percutaneous ablation, radiotherapy, and both locoregional and systemic therapies. Furthermore, the establishment of ICIs-based standard treatments has significantly expanded therapeutic options for advanced HCC, leading to overall improvements in patient prognosis (Nevola et al., 2023; Qin et al., 2023). However, due to the unique characteristics of HCC, the lack of well-defined biomarkers continues to hinder the implementation of precise therapeutic strategies, including targeted therapy and immunotherapy. Progress in HCC treatment still faces substantial challenges posed by the complexity of the tumor microenvironment and mechanisms of therapeutic resistance (Doroshow et al., 2021; Zhou C. et al., 2023).

The development and progression of solid tumors is a complex process, heavily influenced by the biological characteristics of tumor cells and tissues, as well as their interactions with the tumor microenvironment (TME) (de Visser and Joyce, 2023; Elhanani et al., 2023; Hinshaw and Shevde, 2019). In many solid tumors, tumor cells often aggregate into structures known as tumor nests (Boxberg et al., 2019; Jesinghaus et al., 2019; Nakanishi et al., 2001). For example, in hepatocellular carcinoma (HCC), histologically, tumor cells often exhibit trabecular or nest-like growth patterns, where clusters of tumor cells are enveloped by a desmoplastic stroma. The formation of these tumor nests is a key pathological feature associated with the invasive growth and metastatic spread of malignant tumors. Endothelial cells at the margin of tumor nests facilitate tumor cell entry into the bloodstream through fusion with microvessels (Ch’ng et al., 2013). Within the tumor nest, tumor cells can effectively evade attacks from the immune system and mechanical damage caused by blood flow shear stress, due to the protective role of endothelial cells. This protective mechanism helps maintain the stability of the microenvironment within the nest (Ding et al., 2011; Fang et al., 2015). As tumor cells reach distal target organs via the bloodstream, they are able to colonize, proliferate, and establish new metastatic lesions.

In certain malignant tumors, a distinct histological feature is observed in the form of small tumor cell nests, each of which is encapsulated by a layer of peripheral endothelial cells. These configurations give rise to multiple, discrete, and spheroid-like microunits dispersed throughout the tumor tissue (Horn et al., 2006; Jesinghaus et al., 2019; Nakanishi et al., 2001; Zare et al., 2020). HCC, the predominant type of primary liver cancer (PLC), has the ability to metastasize to the portal vein, bile ducts, and liver parenchyma in the form of tumor nests. Notably, during metastasis, tumor nests maintain their structural integrity, with marginal tumor cells exhibiting stem cell-like characteristics (Dong et al., 2021; Reis-Filho et al., 2003). These cells possess tumor-initiating potential, while the central region of the tumor nest displays significant phenotypic heterogeneity among HCC cells. Our previous studies have revealed that a specific F5 cancer-associated fibroblast (F5 CAF) subpopulation, characterized by the expression of COL1A2, COL4A1, COL4A2, CTGF, and FSTL1, which are located at both the center and margin of tumor nests. These F5-CAFs interact with hepatocellular carcinoma (HCC) cells to promote their proliferation and sustain their stemness. (Jing et al., 2024). The spatial heterogeneity of tumor tissues reflects location-specific differences in gene expression, epigenetic regulation, metabolic function, and regenerative capacity. It can reveal both distinct and conserved features of tumor cores and margins, thereby enabling predictions of patient survival and responses to targeted therapies. Furthermore, defining cellular identities and their potential molecular communication networks is critical for advancing our molecular understanding of the tumor ecosystem and for the development of effective therapeutic strategies against solid tumors. (R. Arora et al., 2023; Ben-Moshe and Itzkovitz, 2019; Ma et al., 2022; Shi et al., 2017). However, studies on the spatial association and differences between the central and marginal regions of tumor nests remain limited, and the heterogeneity of these regions has yet to be systematically elucidated. Tumor heterogeneity serves as an important biomarker for assessing the clinical prognosis of cancer patients (Jesinghaus et al., 2017; Kadota et al., 2014; Ma et al., 2019). A deeper understanding of the characteristics of the center-margin ecosystem within tumor nests will provide further insights into the biological features of solid tumors and may offer a crucial theoretical basis for elucidating tumor nest metastasis mechanisms and developing novel therapeutic strategies.

The development of spatial transcriptomics (ST) technology has provided a revolutionary tool for deciphering the spatial heterogeneity of complex tissues (Cheng et al., 2025; Saviano et al., 2020; Ye et al., 2024). ST enables genome-wide RNA quantification within intact tissue sections while preserving precise spatial location information of cells (Fujiwara et al., 2024; Lin et al., 2024; Wang et al., 2022; Zhang et al., 2023), offering a new perspective and technological approach for comprehensively analyzing tumor spatial transcriptomic heterogeneity (Jain and Eadon, 2024; Zhou P. Y. et al., 2023). In this study, we applied ST to systematically identify characteristic gene expression patterns in the marginal and central regions of HCC tumor nests using different methods. The analysis revealed significant differential gene expression among patient samples, while also highlighting conserved gene expression features across different tumor nests. Specifically, metabolic genes were upregulated in the tumor nest center, while immune-related genes were upregulated at the margins. Notably, this characteristic expression pattern may have been established during the early stages of liver fibrous nodule formation. This differential gene expression pattern between the marginal and central regions of the tumor nest reveals spatial heterogeneity in gene expression within tumor tissues, highlighting a previously underappreciated mode of gene regulation within a novel type of tissue unit.

## Materials and methods

### Patients and collection of clinical specimens

**FIGURE 1:**
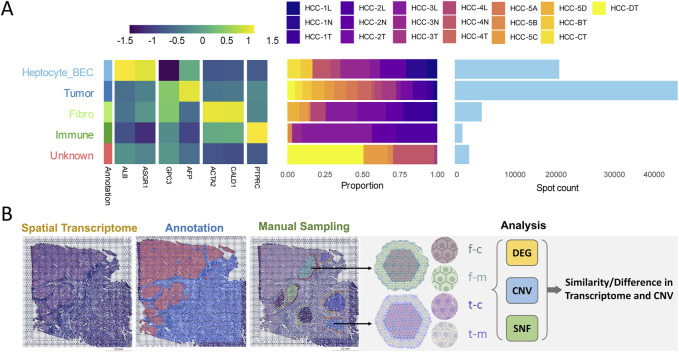
Dimensionality reduction clustering and sampling schematic of ST data. **(A)** Clustering results of ST data. A total of 19 samples (right-top) obtained from 8 HCC patients were included. Hepatocyte_BEC: Hepatocyte-Biliary Epithelial Cells; **(B)** Schematic representation of ST data sampling. f-c, Fibrotic nodule-center; f-m, Fibrotic nodule-margin; t-c, Tumor nest-center; t-m, Tumor nest-margin.

The samples used in this study were obtained from patients with PLC who underwent surgical resection at the Eastern Hepatobiliary Surgery Hospital (EHBH). A total of 25 fresh tissue samples from 11 patients were included for ST analysis. With the joint assistance of at least three pathology experts, we screened samples for the presence of distinct tumor nests based on their characteristic morphological features. As some samples did not exhibit clearly identifiable tumor nests, ultimately, 19 ST samples from 8 HCC patients (Figure 1A) were selected for subsequent research. All patients were randomly selected and provided informed consent. The study protocol was reviewed and approved by the Ethics Committee of the Eastern Hepatobiliary Surgery Hospital.

### Spatial transcriptomics experiments

#### Tissue sample processing

All fresh tissue samples were embedded using optimal cutting temperature (OCT) compound, followed by rapid freezing in pre-cooled isopentane suspended in liquid nitrogen. The samples were subsequently stored at −80°C for future use. Embedding was completed within 30 min of surgical resection for each sample to ensure optimal preservation for cryosectioning.

#### ST experimental workflow

The ST experiment was conducted according to the manufacturer’s guidelines for the Visium Spatial Gene Expression Kit (10x Genomics). Frozen tissue sections (10 μm thick) were mounted onto the 10x Genomics Visium spatial barcode array. During the process, tissues were first fixed in pre-chilled methanol at −20°C for 30 min, followed by H&E staining. Images were then captured using a Leica SCN400 F whole-slide scanner at ×40 magnification. After confirming that the tissue morphology was suitable and RNA integrity was intact (RNA Integrity Number, RIN ≥7), tissue permeabilization and reverse transcription were performed using the Visium Spatial Tissue Optimization Kit (10x Genomics). Following second-strand synthesis and denaturation, the library was constructed and sequencing was carried out using the NovaSeq 6000 sequencing platform (Illumina). Each spot on the array had a diameter of 55 microns, with a center-to-center spacing of 100 microns, covering an area of 6.5 × 6.5 mm^2^. All ST data are archived in the Genomic Sequence Archive (GSA) under the dataset HRA000437, with partial analyses of the TME previously published (Jing et al., 2024; Wu R. et al., 2021).

### Dimensionality reduction and clustering of ST data

All HCC samples in the dataset, consisting of eight patients and 19 samples, were selected for analysis. Preliminary analysis was performed using the Seurat (v5.1.0) (Navarro et al., 2017) package in R (v4.4.0). Gene expression was normalized using the SCTransform function. Multi-sample integration was conducted using the IntegrateData function. For clustering, single-cell data from the Gene Expression Omnibus (GEO) dataset GSE202642 (Zhu et al., 2023) were utilized, following the methods outlined in the original publication. The FindTransferAnchors and TransferData functions were used to integrate single-cell data with the ST data. Dimensionality reduction, clustering, and annotation of major cell types at each spatial location were performed with reference to the literature sources of the ST data.

### ST sampling and analysis

Spatial location clustering was performed using Loupe Browser 8.1.2. The integrity of tumor nests and liver fibrosis nodules was assessed based on hematoxylin and eosin (H&E) staining. Tumor nests or fibrosis nodules with distinct stromal boundaries were considered intact and included in the analysis. Tumor samples lacking well-defined tumor nests were excluded. Based on the analysis, spatial locations corresponding to the margin and center of both tumor nests and fibrosis nodules were selected within Loupe Browser. These selected spatial regions were then imported into R for further analysis. Subsequently, cells within the defined regions were filtered based on the annotations from previous steps.

### Differential gene expression analysis

Differential gene expression analysis was performed at both pseudobulk and spatial location levels in tumor nests and liver fibrosis nodules. At the pseudobulk level, margin and center regions of the same tumor nest or fibrosis nodule were treated as individual units. The AggregateExpression function was used to obtain the pseudobulk expression matrix, allowing for the comparison of expression differences between the margin and center regions across all patient samples. Differential expression at the pseudobulk level was assessed using the DESeq2 method in the FindMarkers function. At the spatial location level, DESeq2 (version 1.44.0) (Love et al., 2014) was applied to identify upregulated and downregulated genes, with genes selected for further analysis if they met the criteria of *p* < 0.05 and |Log2FC| > 0.4. GO analysis was performed using the clusterProfiler package (version 4.12.6) (Wu T. et al., 2021), with pathways displaying a *p*-value <0.05. Gene intersection visualizations were generated using UpSetR (version 1.4.0) (Conway et al., 2017; Lex and Gehlenborg, 2014) and VennDiagram (version 1.7.3) (Chen and Boutros, 2011).

### Transcriptional factors (TF) analysis

We conducted transcription factor enrichment analysis on the identified key genes using the R package RcisTarget (v1.24.0). To ensure robustness, transcription factors with fewer than eight enriched target genes were filtered out prior to downstream interpretation.

### Copy number variation (CNV) analysis

CNV analysis can provide insights into the genomic biological characteristics of tumor cells. To further explore the biological characteristics of tumor cells in different spatial regions within tumor nests, we performed CNV analysis based on ST data in addition to transcriptomic analysis. CNV analysis was performed using the R package inferCNV (version 1.20.0) (Patel et al., 2014; Zhu et al., 2023) to infer copy number variations at the spatial location level. A cutoff value of 0.1 was set for the CNV analysis. CNV scores were calculated by normalizing the corresponding CNV matrix values to a range between −1 and 1, followed by squaring the normalized values. Differences in CNV levels between various sample units were assessed using the Wilcoxon rank-sum test. To compare CNV levels, two-tailed t-tests or Wilcoxon tests were applied, with a significance threshold of *p* < 0.05.

### Clustering analysis

Clustering analysis was performed using the R package SNFtool (version 2.3.1) (Wang et al., 2014) to analyze pseudobulk-level transcriptomic and CNV matrices. A value of K = 20 was selected for SNF function. The resulting clusters were visualized using the R package ggplot2 (version 3.5.1) (Wickham and Wickham, 2016), allowing for a clear graphical representation of the clustering outcomes.

## Results

### Cellular and sample clustering in ST data

The present investigation is centered on HCC, a predominant subtype of PLC, incorporating ST data from 19 samples obtained from 8 HCC patients. Data integration and dimensionality reduction were performed based on universal cell type markers, single-cell datasets, published ST data, and histopathological tissue morphology (details in *Methods*). This approach enabled the identification of the major cell types at spatial locations, including Hepatocyte_BEC, Tumor, Fibroblast, Immune Cell, and Unknown categories (Figure 1A). Subsequent analyses were restricted to spatial spots from tumor regions for tumor nest analysis, while spatial spots from hepatocytes or cholangiocytes were used for the analysis of liver fibrosis nodules. Using standardized tumor nest morphology definitions, histological regions of the liver cancer ST samples were selected for analysis using the Loupe Browser visualization software (Figure 1B). According to the inclusion criteria, tissue samples lacking clear tumor nest structures were excluded. Ultimately, 24 tumor nest regions meeting the criteria were identified from seven tumor ST samples from 3 HCC patients (Supplementary Tables 1, 3). Additionally, 4 ST samples were included, from which 15 liver fibrosis nodule regions were analyzed (Supplementary Tables 2, 3).

### The spatial gene expression patterns of HCC tumor nests exhibit marked heterogeneity

**FIGURE 2:**
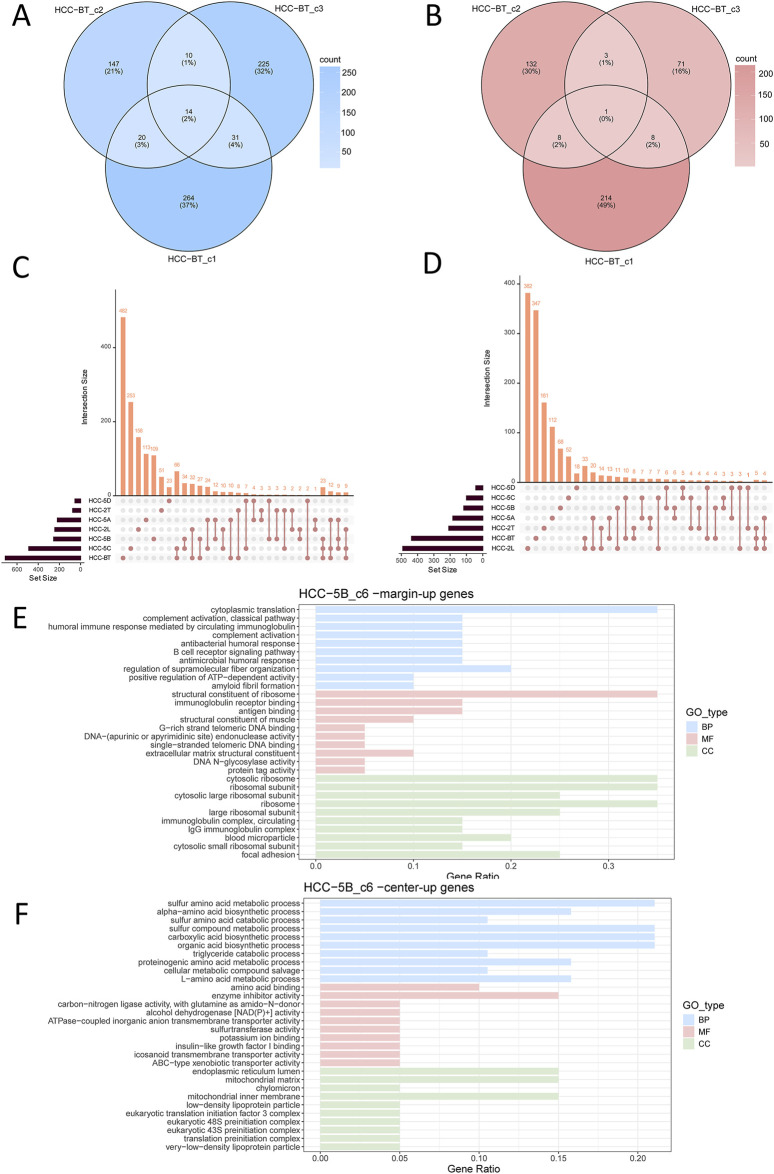
Analysis of differentially expressed genes (DEGs) across tumor nests in HCC patient samples. **(A,B)** Venn diagram showing upregulated DEGs in the marginal **(A)** or central **(B)** regions of three tumor nests from patient HCC-BT; **(C,D)** Upset plot displaying upregulated DEGs in the marginal **(C)** or central **(D)** regions of tumor nests, analyzed at the individual patient level. **(E)** Functional enrichment of genes upregulated in the marginal regions of the tumor nest; **(F)** Functional enrichment of genes upregulated in the central regions of the tumor nest. Enrichment categories include Biological Process (BP), Molecular Function (MF), and Cellular Component (CC).

To systematically characterize the shared transcriptional features between the central and marginal regions of tumor nests, we performed genome-wide differential expression analysis using the DESeq2 package on tumor nest samples from each individual HCC patient. Genes with an adjusted *p*-value <0.05 and |Log2FC|>0.4 were considered significant. This analysis identified 2,226 significantly upregulated and 1,625 significantly downregulated genes (Supplementary Table 1), highlighting pronounced transcriptional differences between central and marginal tumor cells and underscoring the intratumoral heterogeneity within tumor nests. Patient-level comparisons revealed minimal overlap in differentially expressed genes (DEGs) among multiple tumor nests from the same individual (Figures 2A,B). For instance, in the HCC-BT sample, only 2% (14 genes) of upregulated DEGs in the marginal regions were shared among three distinct tumor nests, with nearly no overlap observed in the central regions (Figures 2A,B). Similar results were obtained from another HCC smaple (Supplementary Figures 1A–D), indicating that even within a single patient, different tumor nests exhibit distinct transcriptional profiles. Furthermore, cross-patient comparisons of DEGs revealed only few commonly upregulated genes in the marginal or central regions of tumor nests (Figures 2C,D), suggesting substantial inter-patient heterogeneity in HCC.

To systematically analyze the spatial heterogeneity within tumor nests, we integrated all tumor nest samples and performed a comprehensive comparison of the gene expression profiles between the central and marginal regions. Using R software, we constructed upset plots to illustrate the overlap and unique sets of DEGs across these tumor nest regions. Notably, among the upregulated genes in the tumor nest margin, samples HCC-BT_c1, HCC-BT_c2, HCC-2T-c1, HCC-5C_c3, and HCC-2L_c2 exhibited several DEGs. Similarly, among the downregulated genes in the tumor nest center, samples HCC-5B_c1, HCC-2L_c2, HCC-BT_c2, HCC-BT_c3, HCC-BT_c1, and HCC-5C_c3 demonstrated considerable differential expression. Notably, the tumor nest samples from HCC-BT, HCC-5C, and HCC-2L exhibited both significantly upregulated and downregulated genes, which may reflect the larger number of analyzed samples. However, few commonly upregulated or downregulated genes were shared across all samples (Supplementary Figures 2A,B). This finding was further corroborated by the DEG upset plot analysis grouped by patient (Figures 2C,D), indicating significant transcriptomic heterogeneity among the tumor nests.

### The spatial gene expression patterns of HCC tumor nests exhibit certain shared characteristics

Despite the substantial heterogeneity observed both among patients and among tumor nests within individual samples, a subset of overlapping genes was still identified (Supplementary Figures 2A,B). Functional enrichment analysis of the upregulated and downregulated genes in each patient sample revealed intriguing spatial trends. Specifically, genes upregulated in the marginal regions of tumor nests were predominantly enriched in immune-related pathways, including B cell receptor signaling pathway and antigen binding (Figure 2E). In contrast, genes upregulated in the central regions were mainly associated with metabolic processes (Figure 2F). These spatially distinct enrichment patterns were consistently observed across multiple patient samples (Supplementary Figure 3). Together, these findings suggest that, despite the high degree of heterogeneity, there are conserved spatial transcriptional features across tumor nests. Namely, marginal tumor nest cells tend to upregulate genes involved in immune regulation, whereas central tumor nest cells preferentially express genes related to proliferation and metabolism.

### Spatial gene expression heterogeneity between fibrotic nodules and patient samples

Dysplastic nodules (DNs), as critical precancerous lesions, exhibit a high incidence in patients with liver fibrosis. Epidemiological data indicate that approximately 90% of HCC cases arise on a background of liver fibrosis (Gazelakis et al., 2021). Both low-grade and high-grade DNs represent key precancerous stages during the progression of liver fibrosis and harbor a potential risk for transformation into HCC (Di Tommaso et al., 2013). Based on these observations, we sought to investigate whether the gene expression characteristics in the marginal and central regions of fibrotic nodules are potentially associated with those found in HCC tumor nests.

**FIGURE 3:**
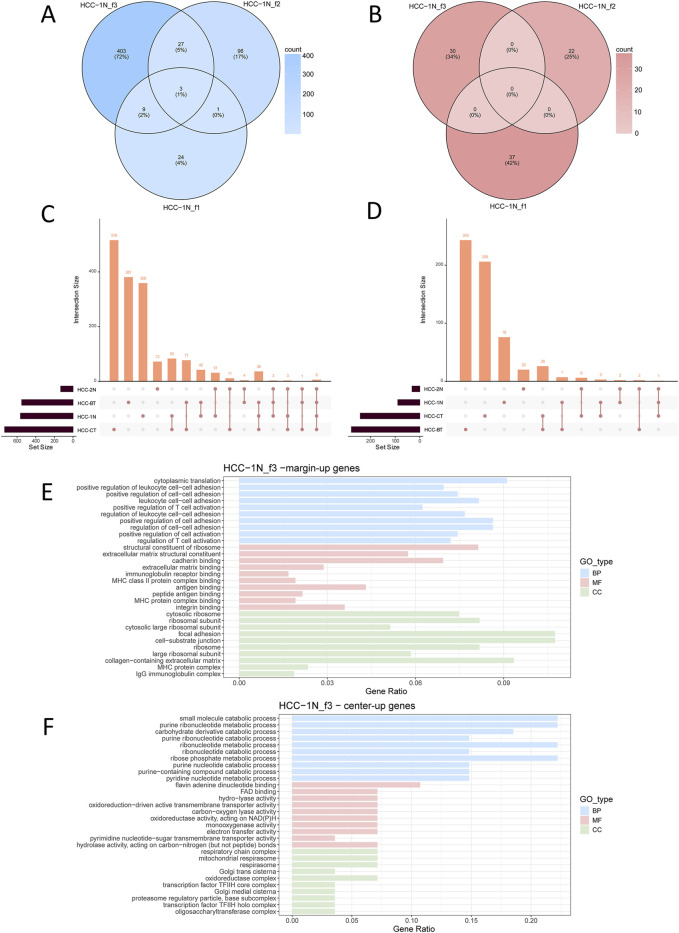
Differential gene expression analysis of fibrotic nodules in individual patients. Shown are the results of the representative sample HCC-1N. See also Supplementary Figure 4. **(A,B)** Venn diagram showing upregulated DEGs in the marginal **(A)** or central **(B)** regions of three fibrotic nodules from patient HCC_1N; **(C,D)** Upset plot displaying upregulated DEGs in the marginal **(C)**or central **(D)** regions of fibrotic nodules, analyzed at the individual patient level. **(E)** Functional enrichment of upregulated genes in the marginal regions of fibrotic nodules; **(F)** Functional enrichment of upregulated genes in the central regions of fibrotic nodules. Enrichment analyses were categorized into Biological Process (BP), Molecular Function (MF), and Cellular Component (CC).

Analysis ultimately resulted in the identification of 2,231 significantly upregulated genes and 675 significantly downregulated genes (Supplementary Table 2). At the patient level, differential expression analysis was performed to compare the gene expression profiles at the margin and center of individual fibrotic nodules. The results showed that, within each patient, the overlap of DEGs between distinct fibrotic nodules was minimal (Figures 3A,B; Supplementary Figures 4A–F). Subsequently, the DEGs from fibrotic nodules of all patients were integrated and subjected to gene interaction network analysis. Although a set of six central interacting genes, commonly upregulated in the marginal regions of fibrotic nodules, was identified in a subset of patients (e.g., HCC_BT), overall the fibrotic nodules exhibited significant heterogeneity (Supplementary Figures 5A,B). This observation was further validated when the differential expression analysis was stratified by patient (Figures 3C,D). Moreover, integrated interaction network analysis of the upregulated and downregulated genes in fibrotic nodules yielded results similar to those observed in tumor nests: the nodule margin predominantly enriched for immune regulatory pathways, whereas the center was mainly associated with metabolic pathways (Figures 3E,F). This conclusion was consistently confirmed across multiple patient samples (Supplementary Figure 6).

### Functional analysis of DEGs between the center and margin of tumor nests

**FIGURE 4:**
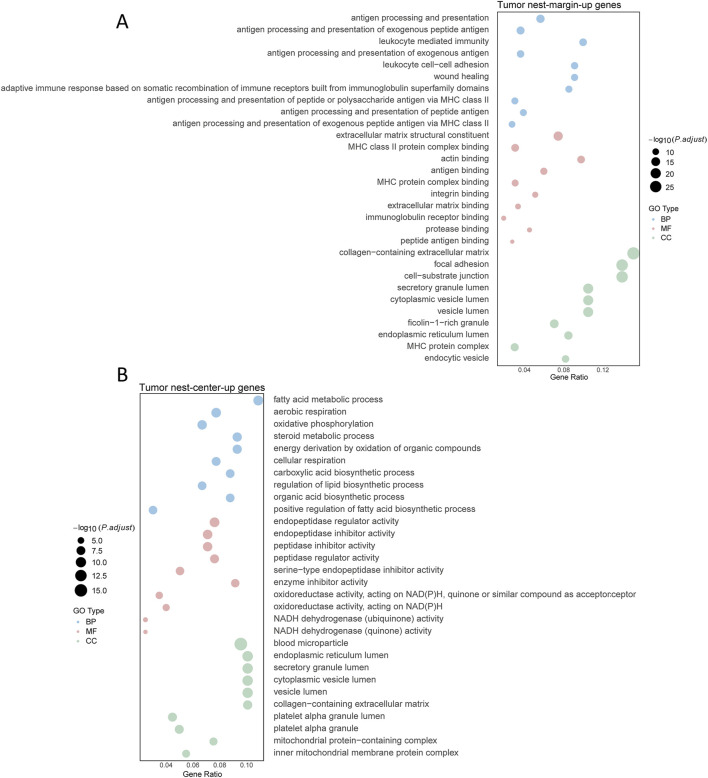
Functional enrichment analysis of differential genes in tumor nests. **(A)** Functional enrichment analysis of upregulated genes in the marginal region of tumor nests; **(B)** Functional enrichment analysis of upregulated genes in the central region of tumor nests. Enrichment analyses are categorized into Biological Process (BP), Molecular Function (MF), and Cellular Component (CC).

Due to the substantial heterogeneity observed both among tumor nests and among patients, coupled with the large number of samples analyzed, it was challenging to identify a sufficient number of commonly upregulated or downregulated DEGs across distinct tumor nests. To address this, we established a selection criterion whereby a gene was deemed a characteristic tumor nest-specific differential gene if it was consistently expressed in at least two independent tumor nest samples. Using this criterion, we ultimately identified 354 significantly upregulated interaction genes and 202 significantly downregulated interaction genes in the tumor nest center. KEGG pathway enrichment analysis of these characteristic interaction genes revealed that genes (MFGE8, SNX20, FBXO38, EMP3, etc.) highly expressed in the tumor nest margin were significantly enriched in immune regulatory pathways, cytokine—including cytokine receptor interactions, and antigen presentation—whereas genes (SLC35G2, TOP1MT, GGT7, MAIP1, etc.) highly expressed in the tumor nest center were mainly associated with metabolic pathways—glycolysis, the tricarboxylic acid cycle, and oxidative phosphorylation (Figures 4A,B). These findings are consistent with the overall analysis presented earlier (Figure 2), confirming that the tumor nest margin is predominantly enriched in immune regulatory pathways, while the tumor nest center is primarily enriched in metabolic pathways.

We performed transcription factor (TF) enrichment analysis on the identified key genes and found that TFs such as HAND1, RELA, and CLOCK were upregulated in the tumor margin, whereas members of the zinc finger family were predominantly upregulated in the tumor center (Supplementary Figure 7). Consistent with our findings, RELA is a key component of the NF-κB signaling pathway, which plays a critical role in regulating immune responses. In contrast, zinc finger transcription factors are known to be involved in the regulation of gene transcription and may be associated with enhanced protein synthesis required for cellular proliferation and metabolic activity.

Taken together, these results indicate that, despite the pronounced transcriptomic heterogeneity both among tumor nests within individual patients and among patients, distinct gene expression differences persist between the tumor nest center and margin. Specifically, cells in the margin exhibit significantly elevated expression of genes associated with immune regulation, whereas cells in the center primarily express genes related to metabolic processes.

### Differences in CNV levels between tumor nests and fibrotic nodules

**FIGURE 5:**
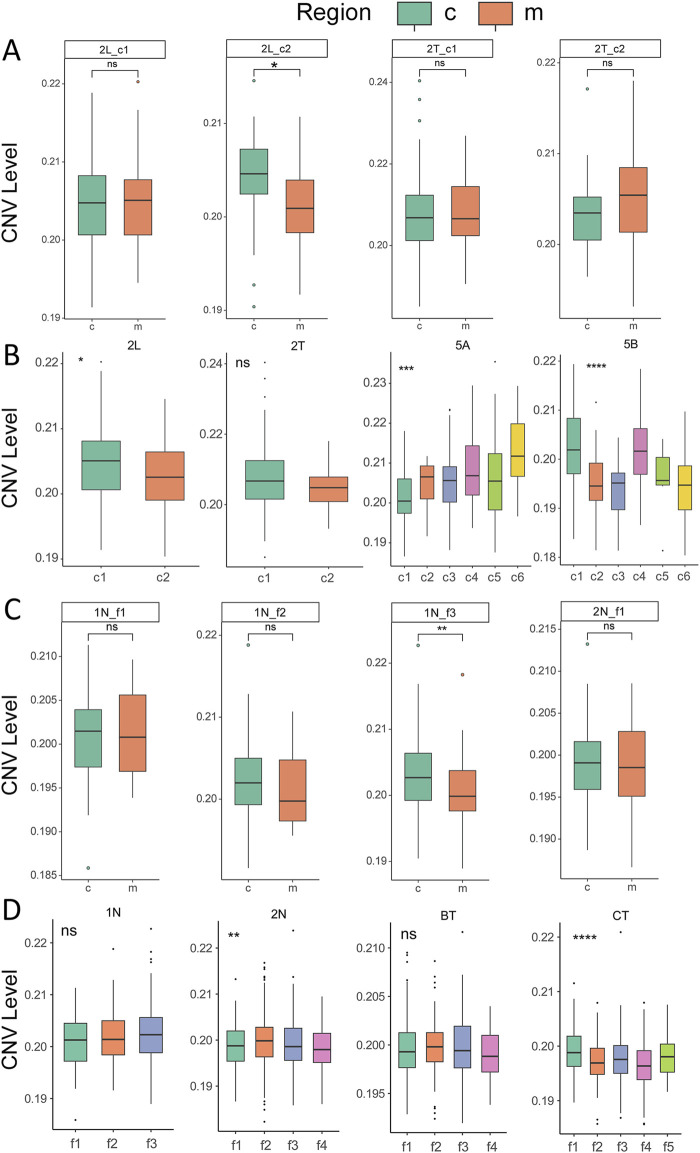
Comparison of CNV levels in tumor nests and fibrotic nodules. **(A)** Comparison of CNV levels between the central and marginal regions of tumor nests. c, center; m, margin; **(B)** Overall CNV levels across different tumor nests within the same sample. **(C)** Comparison of CNV levels between the central and marginal regions of fibrotic nodules. c, center; m, margin; **(D)** Overall CNV levels across different fibrotic nodules within the same sample. *, **, ***, **** indicate, *p* < 0.05, *p* < 0.01, *p* < 0.001, *p* < 0.0001, respectively.

To investigate the biological characteristics associated with genetic alterations in tumor nests, CNV analysis was performed using the inferCNV package, with tumor nests as the units of analysis. The results indicated that the CNV levels between the central and marginal regions of most tumor nests did not exhibit statistically significant differences (Figure 5A; Supplementary Figure 8A), suggesting that clonal evolution within a single tumor nest is not pronounced. Further analysis, integrating CNV characteristics across all tumor nests within individual patients, revealed significant CNV differences among different tumor nests within the same patient in samples such as HCC-2L, HCC-2T, HCC-5C, and HCC-5D (Figure 5B; Supplementary Figure 8B). This finding highlights the intratumoral heterogeneity and evolutionary diversity of tumor nests. Similar patterns were observed in fibrotic nodules, where the CNV levels between the central and marginal regions of the nodules did not show statistically significant differences. However, substantial variations were present among different fibrotic nodules within the same patient (Figures 5C,D; Supplementary Figure 9). These observations further underscore the complexity and heterogeneity present in both tumor nests and fibrotic nodules.

### Comparison of tumor nests and fibrotic nodules at the pseudobulk level

To further explore the overall patterns of differential gene expression between tumor nests and fibrotic nodules, and their respective central and marginal regions, and to account for the sparsity of the raw single-cell data, we performed pseudobulk analysis for each tumor nest and fibrotic nodule. DEGs between the central and marginal regions were identified, revealing distinct sets of DEGs unique to each region (Supplementary Figure 10A). Functional enrichment analysis of the DEGs within tumor nests yielded results similar to previous findings: marginal cells exhibited significantly higher expression of genes associated with immune regulation, while central cells were enriched in genes related to metabolic processes (Supplementary Figures 10B,C).

**FIGURE 6:**
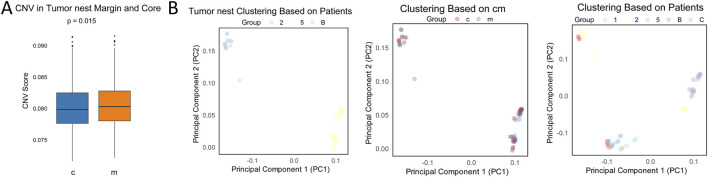
Comparison of transcriptomic and CNV profiles at the pseudobulk level. **(A)** Comparison of CNV at the pseudobulk level in tumor nests. c, center; m, margin; **(B)** Clustering of transcriptomic similarity at the pseudobulk level across tumor nests and analysis units. c, center; m, margin.

At the pseudobulk level, comparison of CNV revealed that tumor nest marginal regions had higher CNV levels, while the central regions exhibited lower CNV levels (Figure 6A). In contrast, fibrotic nodules showed higher CNV levels in the central regions and lower CNV levels in the marginal regions (Supplementary Figure 11A). To investigate the similarities in transcriptional and CNV profiles between tumor nests and fibrotic nodules at the central and marginal regions, we employed a similarity network fusion (SNF) model that incorporated both CNV score matrices and pseudobulk-level transcriptomic data. Regardless of the tissue type, all analysis units clustered by patient, with no significant differences observed between regions within the same patient. Central and marginal samples did not separate into distinct clusters (Figure 6B; Supplementary Figures 11B,C), suggesting that patient-specific heterogeneity predominates over regional heterogeneity within tumor nests. This finding also helps explain the limited overlap of DEGs both within tumor nests and across patients (Figures 2, 3; Supplementary Figures 1, 4).

## Discussion

Studies on the division of labor among cancer cells within tumor remain largely unreported in current literature (Lim et al., 2017; Tammela et al., 2017; S. Zhang et al., 2023). ST has demonstrated remarkable potential in advancing our understanding of HCC and accelerating drug discovery (Ståhl et al., 2016). By enabling the visualization of gene expression across spatial dimensions within tissues or organs, ST reveals the intricate architecture of the tumor microenvironment (Chung et al., 2022; Liu et al., 2023). This spatial resolution allows for more precise identification of potential drug targets and resistance mechanisms, as well as the elucidation of immunotherapeutic targets. Collectively, these capabilities position ST as a powerful tool for driving the development of personalized medicine in HCC (Requena et al., 2024; Zhang et al., 2023). Our study, based on ST technology, compares the differences in transcriptomic profiles and CNV between the central and marginal regions of HCC tumor nests and fibrotic nodules leads to several key conclusions: marginal tumor nest cells are primarily involved in immune regulation, whereas central cells are more closely associated with growth and metabolism. This spatial gene expression architecture may be established as early as the fibrotic nodule stage. The observed spatial expression patterns suggest that marginal tumor nest cells might engage in immune escape by activating immune regulatory pathways through close interactions with immune cells and the TME (Such as the gene MFGE8, which is a potent pro-angiogenic factor. It can promote the secretion of VEGF and endothelin-1 (ET-1) by mesenchymal stem cells, enhance M2 polarization of tumor-associated macrophages, and thereby facilitate tumor angiogenesis and tumor growth (Pei et al., 2025; Raymond et al., 2009). Conversely, central tumor nest cells likely redirect metabolic processes to support rapid cell proliferation (Such as the gene TOP1MT, which encodes the human mitochondrial DNA topoisomerase I. It promotes tumor cell growth and proliferation by regulating mitochondrial gene translation and energy metabolism.) (Pommier et al., 2022; Rossi et al., 2022; Sivanand et al., 2024). Additionally, it is important to note that these functional characteristics of marginal tumor nest and fibrotic nodule cells may be linked to their direct interactions with a greater number of immune cells.

Our comparative analysis revealed distinct clonal evolutionary differences between the central and marginal regions of tumor nests and fibrotic nodules (Figure 5; Supplementary Figures 8, 9). Previous studies have demonstrated a significantly higher burden of somatic mutations in fibrotic nodules compared to normal liver tissue (Brunner et al., 2019), suggesting that tumor-associated mutational patterns may already emerge during the fibrotic stage. However, our findings reveal that the CNV landscape within fibrotic nodules is distinct from that observed in tumor nests. While such differences were minimal and variable at the individual tumor nest or fibrotic nodule level in sparse data, CNV changes at the multi-sample and multi-patient levels were generally lower in the central regions of tumor nests compared to the marginal regions (Figure 6A). In contrast, fibrotic nodules exhibited higher overall CNV changes in their central regions relative to the margin. This divergence may be attributed to the differential microenvironmental pressures faced by these regions: tumor nests are subjected to immune-mediated selection, whereas fibrotic nodules are primarily involved in tissue repair and regeneration (Ally et al., 2017). These contrasting functional roles likely contribute to the emergence of distinct mutational patterns. Notably, tumor nests exhibit a unique spatial distribution of evolutionary clones, accompanied by more intense mutational dynamics, which may underlie the differences in CNV expression profiles between tumor nests and fibrotic nodules.

Due to technical limitations, it has traditionally been challenging to conduct a systematic comparison of the transcriptomic and genomic features between the marginal and central regions of tumor nests. ST technology offers an excellent platform for this type of analysis. Unfortunately, due to the spatial resolution limitations of the ST technology used in this study, such as relatively large capture spots that preclude single-cell resolution, inability to resolve subcellular structures, limited spatial precision, and challenges in distinguishing gene expression differences between adjacent cells—certain aspects of spatial heterogeneity remain difficult to characterize (Bonev et al., 2024; Jin et al., 2024). Consequently, our sample cohort did not adequately capture the interactions within the tumor nest (data not shown). However, with the advancement of higher-resolution ST, this issue will be addressed in future studies.

In conclusion, this study provides a comprehensive analysis of the gene expression profiles and CNV characteristics in the central and marginal regions of HCC tumor nests. The findings offer molecular-level evidence for the spatial heterogeneity of liver cancer and reveal the biological logic underlying the functional organization of tumor nests. The center-margin division within tumor nests may present a novel strategy for targeted metabolic and immune combination therapies. Understanding the spatial heterogeneity of disease onset and progression is one of the key steps in optimizing the presentation of disease-specific molecular interaction maps (Zhang et al., 2022). This knowledge refines the spatial characteristics of tumor architecture, not only revealing the gene expression features of tumor nest units, but also indicating a potential association with fibrotic nodules. These findings may provide novel molecular evidence and guidance for the early identification and intervention of the progression from liver cirrhosis to hepatocellular carcinoma (Arora et al., 2023).

## Data Availability

The datasets presented in this study can be found in online repositories. The names of the repository/repositories and accession number(s) can be found in the article/supplementary material.
