# Supplementary material for: Comparative transcriptomic and genomic analysis of tumor cells in the marginal and center regions of tumor nests in human hepatocellular carcinoma
Source: Front Cell Dev Biol. 2025 Jul 16;13:1611951. doi: 10.3389/fcell.2025.1611951 (PMC12307378; doi:10.3389/fcell.2025.1611951)
Supplement: Supplementary file 1 [file DataSheet1.pdf]

Supplementary Material

Comparative Transcriptomic and Genomic Analysis of Tumor Cells in the Marginal and Center Regions of Tumor Nests in Human Hepatocellular Carcinoma

Ziyi Li[1,5,†], Yikai Hu[2,5,†], Zhuotian He[3], Heyi Xu[4], Hongyang Wang[1,2,5]*, Yufei He[6]*

[1] Institute of Metabolism and Integrative Biology, Fudan University, Shanghai, China

[2] Fudan University Shanghai Cancer Center, Department of Oncology, Shanghai Medical College, Fudan University, Shanghai, China

[3] High School Affiliated to Shanghai Jiao Tong University, Jiading Campus, Shanghai, China

[4] Ulink college of Shanghai, Shanghai, China

[5] National Center for Liver Cancer and International Cooperation Laboratory on Signal Transduction, Eastern Hepatobiliary Surgery Institute/Hospital, Shanghai, China

[6] Molecular Pathology Laboratory, National Center for Liver Cancer, Eastern Hepatobiliary Surgery Hospital, Shanghai, China

[†] These authors contributed equally to this work.

* Correspondence.

E-mail addresses: yfhe@sibcb.ac.cn (Yufei He), and hywangk@vip.sina.com (Hongyang Wang)

Supplementary Figures

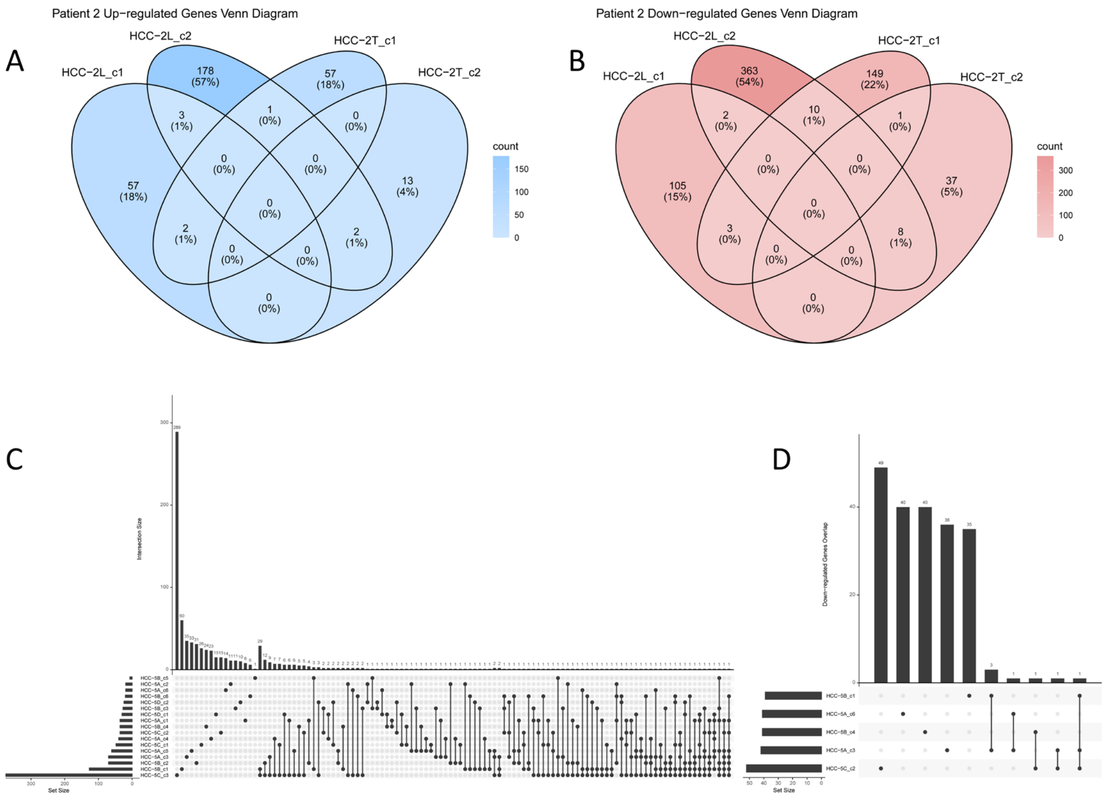

Figure S1. Differential gene analysis of tumor nests in patient samples. (A, B) Venn diagram analysis of upregulated differential genes in the marginal (A) or central (B) region of four tumor nests from Patient-2 sample (HCC_2); **(C, D)** Upset plot of upregulated genes in the marginal (C) or central (D) region of the tumor nest from Patient-5.

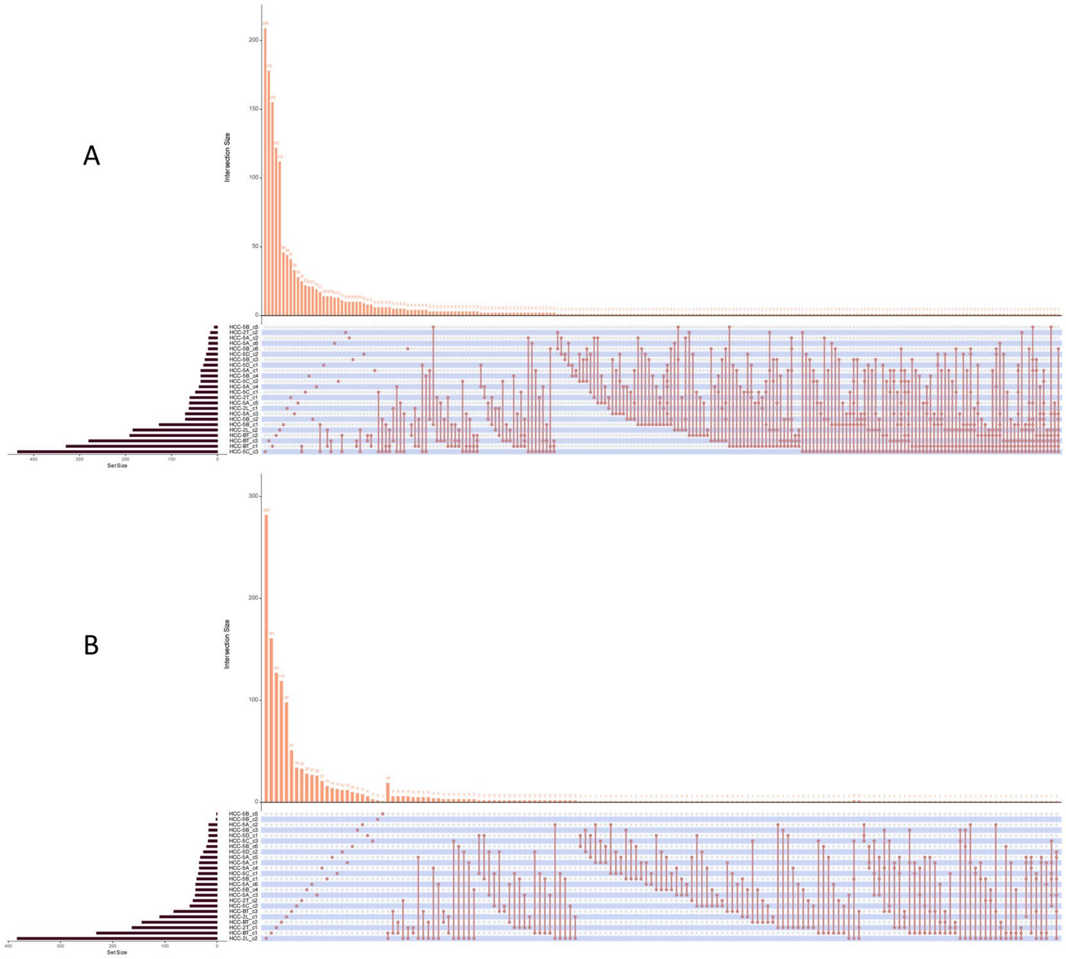

Figure S2. Differential gene analysis of tumor nest samples. (A) Upset plot of upregulated genes in the marginal region of all tumor nest samples; **(B)** Upset plot of upregulated genes in the central region of all tumor nest samples.

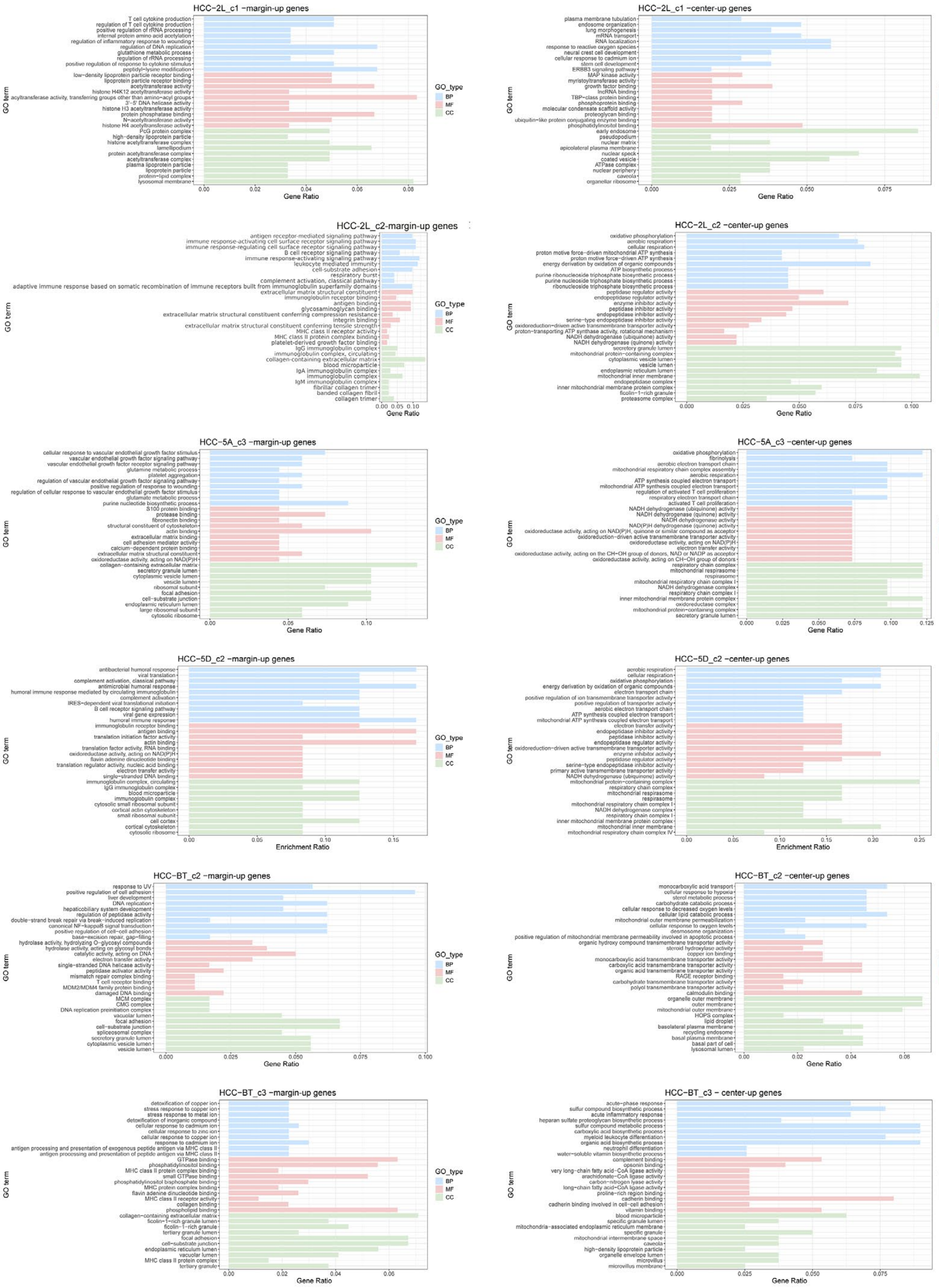

Figure S3. Functional enrichment analysis of tumor nest differential genes. Left: Functional enrichment analysis of upregulated genes in the marginal region of different tumor nests; **Right:** Functional enrichment analysis of upregulated genes in the central region of different tumor nests; BP: Biological Process; MF: Molecular Function; CC: Cellular Component.

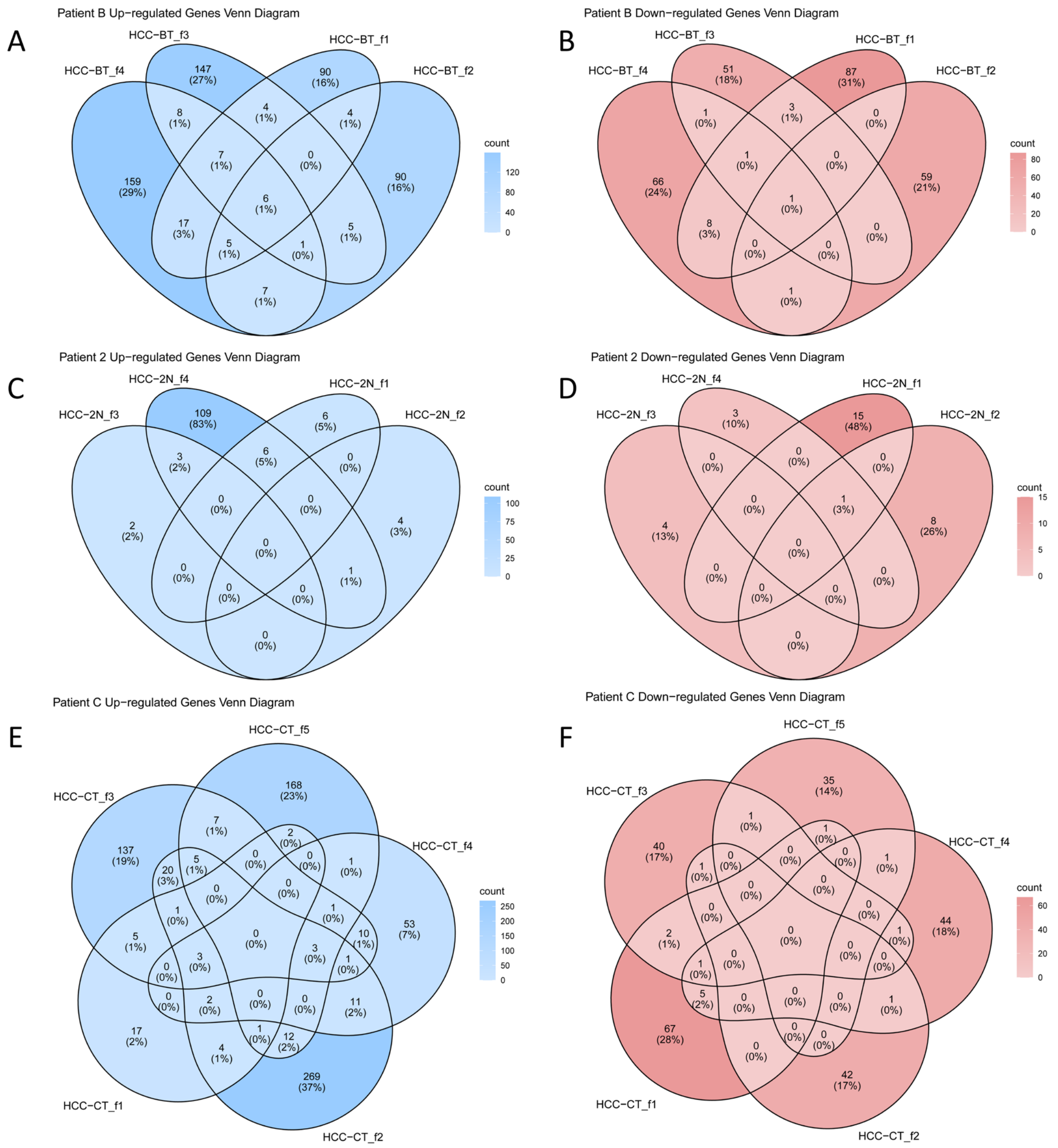

Figure S4. Differential gene analysis of fibrotic nodules in patient samples. (A,B) Venn diagram showing upregulated DEGs in the marginal (A) or central (B) regions of four fibrotic nodules from patient HCC-BT; **(C, D)** Venn diagram showing upregulated DEGs in the marginal (C) or central (D) regions of four fibrotic nodules from patient HCC-2N; **(E, F)** Venn diagram showing upregulated DEGs in the marginal (E) or central (F) regions of five fibrotic nodules from patient HCC-CT.

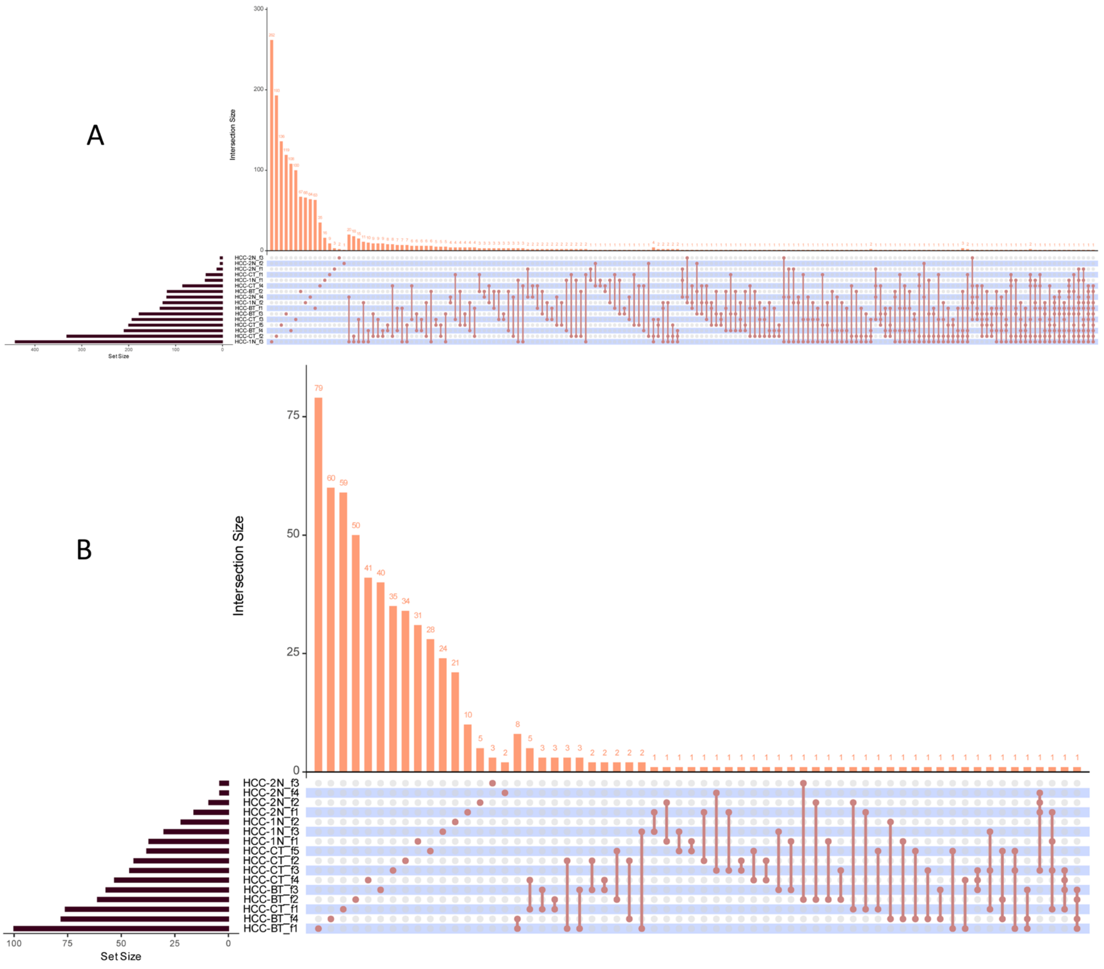

Figure S5. Differential gene analysis of fibrotic nodule samples. **(A)** Upset plot of upregulated genes in the marginal region of all fibrotic nodule samples; **(B)** Upset plot of upregulated genes in the central region of all fibrotic nodule samples.

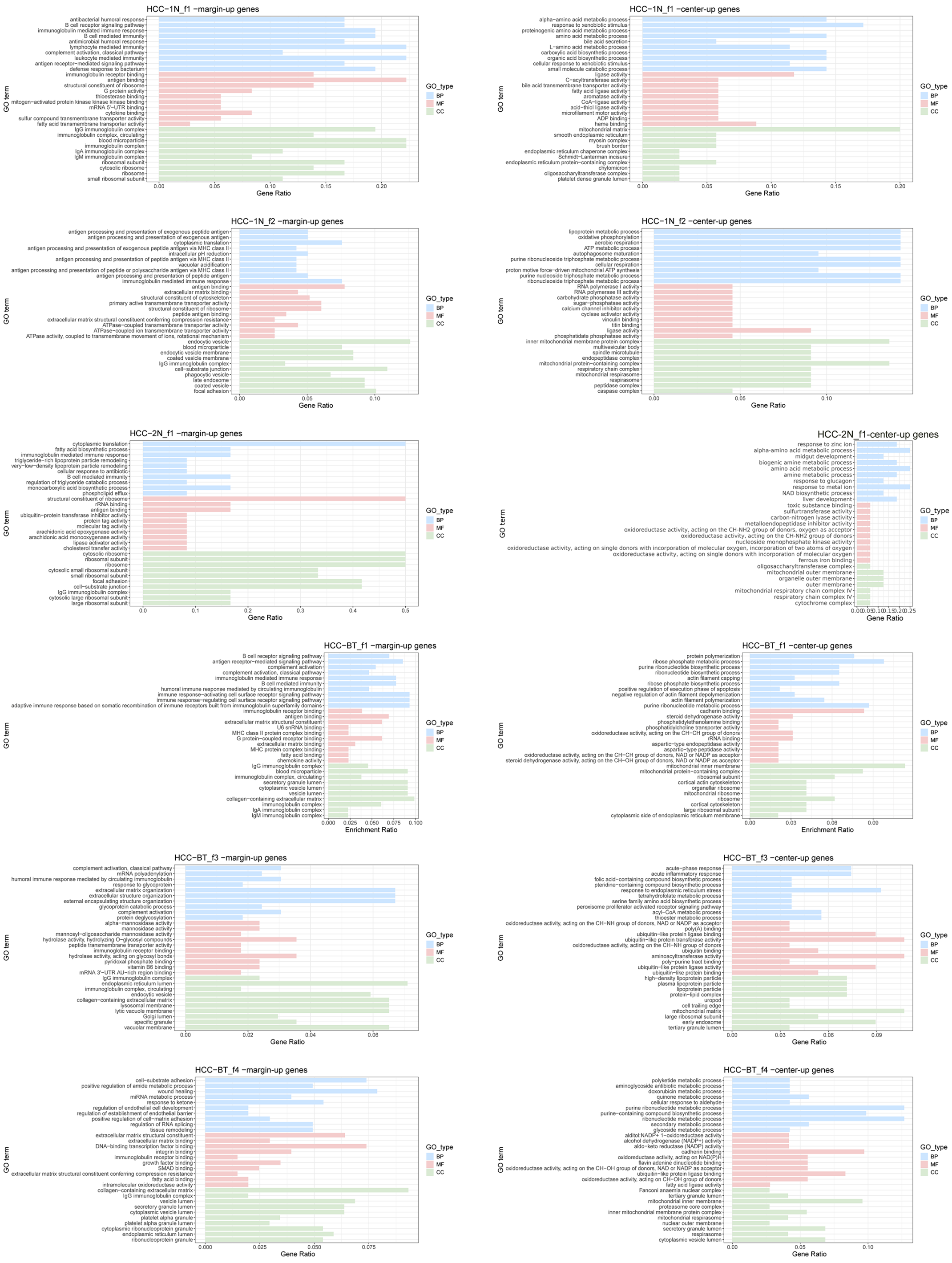

Figure S6. Functional enrichment analysis of fibrotic nodule differential genes. **Left:** Functional enrichment analysis of upregulated genes in the marginal region of fibrotic nodules; **Right:** Functional enrichment analysis of upregulated genes in the central region of fibrotic nodules; BP: Biological Process; MF: Molecular Function; CC: Cellular Component.

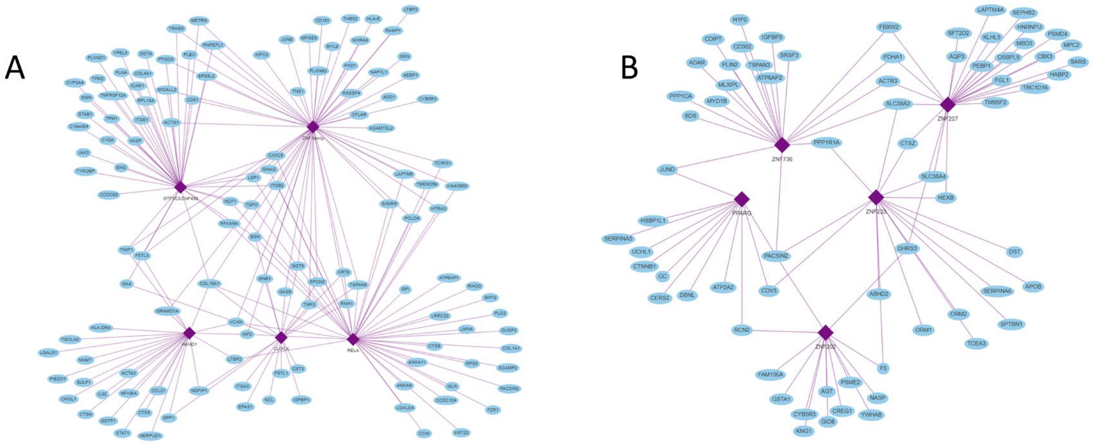

Figure S7. Transcriptional regulatory networks predicted based on key genes. (A) The top five transcription factors, ranked by normalized enrichment score (NES), were predicted based on key genes that are upregulated at the margin of the tumor nest; **(B)** The top five transcription factors, ranked by NES, were predicted based on key genes that are upregulated at the margin of the tumor nest.

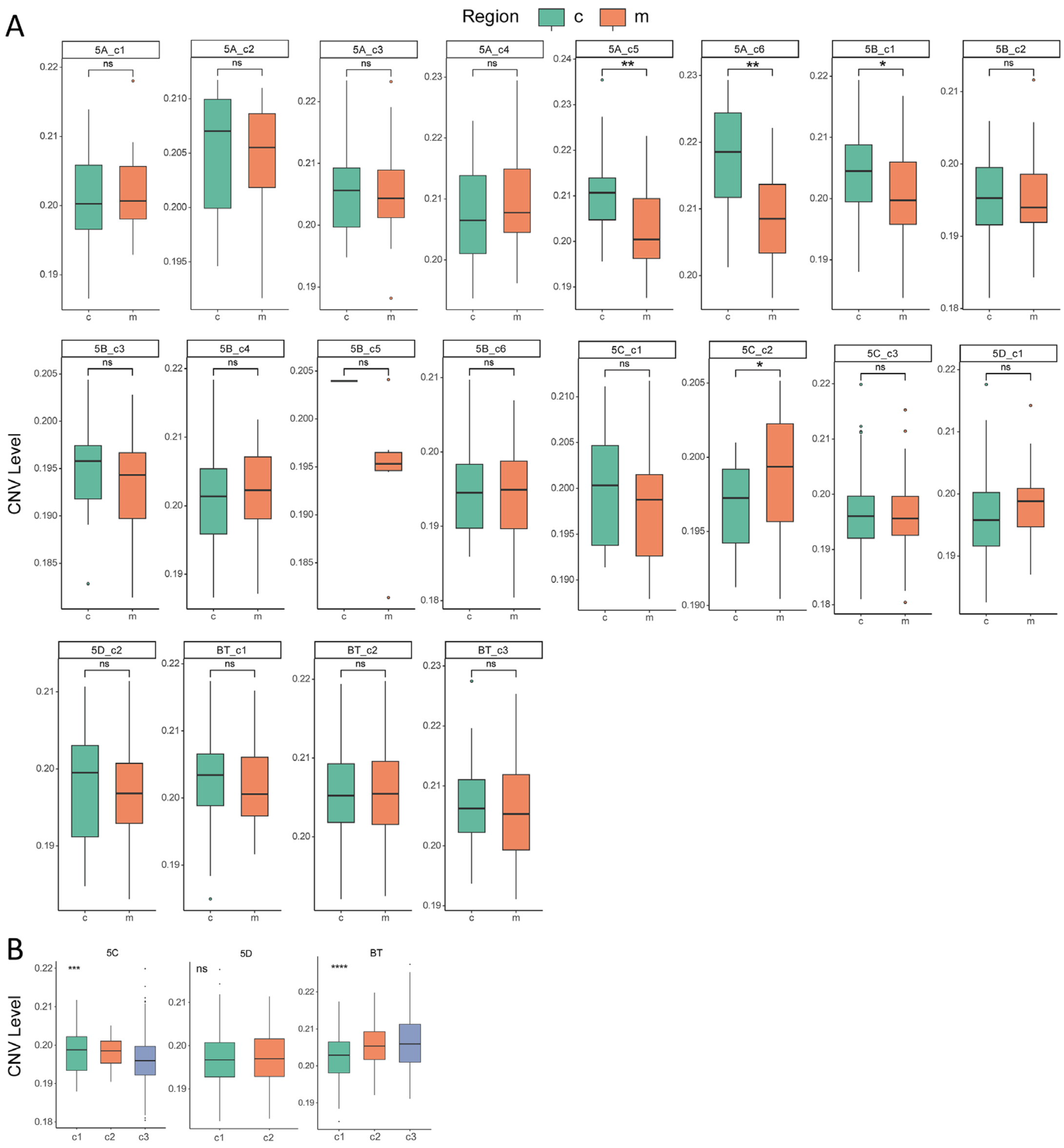

Figure S8. Comparison of CNV levels in tumor nests. (A) Comparison of CNV levels between the central and marginal regions of tumor nests. Abbreviations: c: center; m: margin; **(B)** Overall CNV levels within the tumor nest of the same sample. *, **, ***, **** indicate, $p < 0.05$, $p < 0.01$, $p < 0.001$, $p < 0.0001$, respectively.

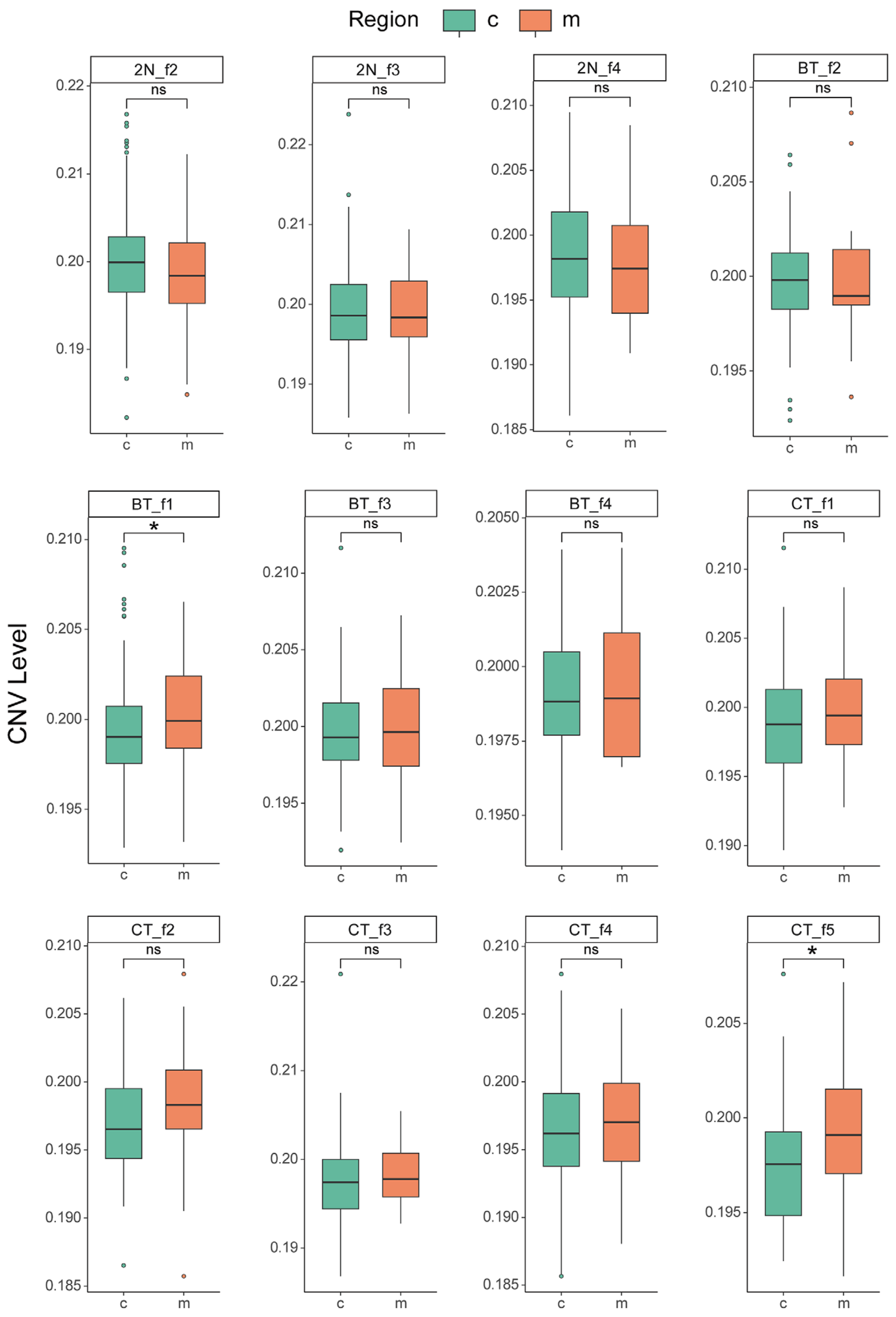

Figure S9. Comparison of CNV levels in the central and marginal regions of fibrotic nodules.

Abbreviations: c: center; m: margin. *, **, ***, **** indicate, $p < 0.05$, $p < 0.01$, $p < 0.001$, $p < 0.0001$, respectively.

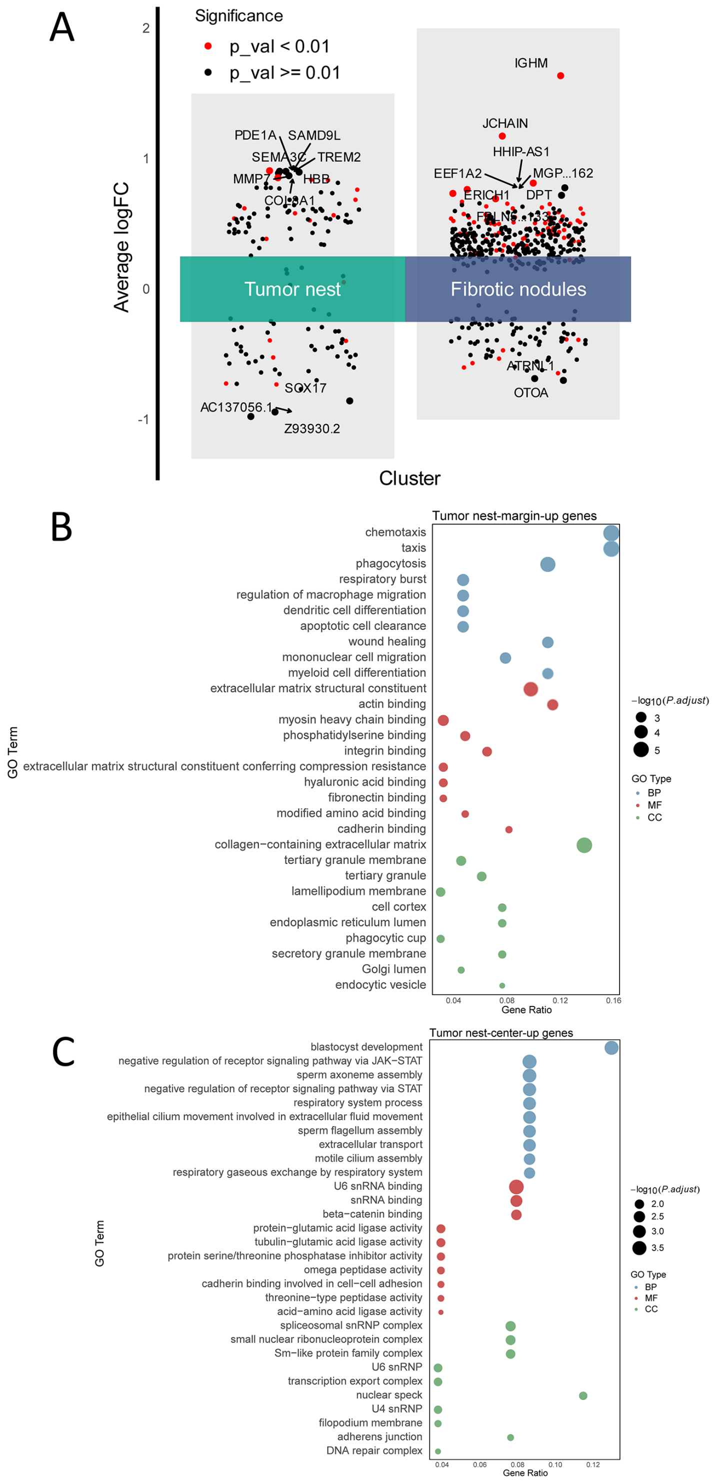

Figure S10. Differential genes expression and pseudobulk-level functional enrichment analysis. **(A)** Volcano plot of differential genes across different samples. Points represent differential genes; the top 10 genes based on |log2FC| are highlighted and labeled; **(B, C)** Gene Ontology (GO) enrichment analysis showing upregulated DEGs in the marginal (B) or central (C) regions of tumor nests at the pseudobulk level. BP: Biological Process; MF: Molecular Function; CC: Cellular Component.

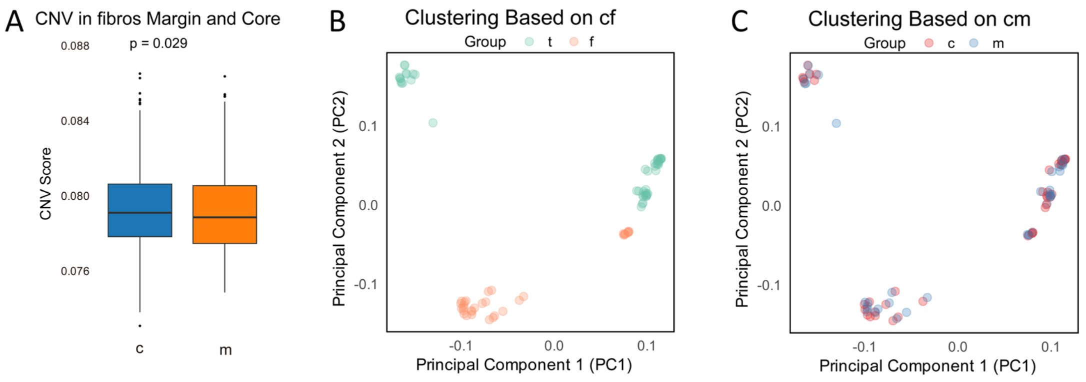

Figure S11. Comparison of transcriptomic and CNV levels at the pseudobulk level. (A) Comparison of CNV levels at the pseudobulk level in fibrotic nodules;. **(B)** Clustering analysis between different analysis units; Abbreviations: t: tumor nest; f: fibroblast; **(C)** Clustering of the marginal and central regions of tumor nests and fibrotic nodules. Abbreviations: c: center; m: margin.

Supplementary Tables

Table S1. Statistics of upregulated and downregulated genes in tumor nests

Samples	Nests	Total Count	up_Count	down_Count
HCC-2L	HCC-2L_c1	172	62	110
HCC-2L	HCC-2L_c2	567	184	383
HCC-2T	HCC-2T_c1	223	60	163
HCC-2T	HCC-2T_c2	61	15	46
HCC-5A	HCC-5A_c1	70	36	34
HCC-5A	HCC-5A_c2	35	19	16
HCC-5A	HCC-5A_c3	112	70	42
HCC-5A	HCC-5A_c4	74	40	34
HCC-5A	HCC-5A_c5	93	61	32
HCC-5A	HCC-5A_c6	60	19	41
HCC-5B	HCC-5B_c1	166	127	39
HCC-5B	HCC-5B_c2	72	70	2
HCC-5B	HCC-5B_c3	43	27	16
HCC-5B	HCC-5B_c4	77	36	41
HCC-5B	HCC-5B_c5	8	7	1
HCC-5B	HCC-5B_c6	40	20	20
HCC-5C	HCC-5C_c1	84	48	36
HCC-5C	HCC-5C_c2	88	36	52
HCC-5C	HCC-5C_c3	452	435	17
HCC-5D	HCC-5D_c1	46	30	16
HCC-5D	HCC-5D_c2	50	24	26
HCC-ST2	HCC-BT_c1	560	329	231
HCC-ST2	HCC-BT_c2	335	191	144
HCC-ST2	HCC-BT_c3	363	280	83

Table S2. Statistics of upregulated and downregulated genes in fibrotic nodules

Samples	Nodules	Total Count	up_Count	down_Count
HCC-1N	HCC-1N_f1	74	37	37
HCC-1N	HCC-1N_f2	149	127	22
HCC-1N	HCC-1N_f3	472	442	30
HCC-2N	HCC-2N_f1	28	12	16
HCC-2N	HCC-2N_f2	14	5	9
HCC-2N	HCC-2N_f3	9	5	4
HCC-2N	HCC-2N_f4	123	119	4
HCC-ST2	HCC-BT_f1	233	133	100
HCC-ST2	HCC-BT_f2	14	5	9
HCC-ST2	HCC-BT_f3	9	5	4
HCC-ST2	HCC-BT_f4	288	210	78
HCC-ST3	HCC-CT_f1	111	35	76
HCC-ST3	HCC-CT_f2	376	332	44
HCC-ST3	HCC-CT_f3	239	193	46
HCC-ST3	HCC-CT_f4	138	85	53

Table S3. Information of patients

Patients	Age	Sex	Ethnicity	Etiology	MVI	TNM Groups	Cirrhosis
HCC-1	54	Male	East Asia	HBV	M1	T2N0M0	Yes
HCC-2	55	Female	East Asia	HBV	M2	T4N0M0	No
HCC-3	61	Male	East Asia	Non-HBV	M2	T2N0M0	No
HCC-4	60	Male	East Asia	HBV	M0	T1bN0M0	No
HCC-5	52	Female	East Asia	HBV	M0	T1aN0M0	Yes
HCC-ST2	42	Male	East Asia	HBV	M2	T1bN0M0	Yes
HCC-ST3	55	Female	East Asia	HBV	M1	T3N0M0	Yes
HCC-ST4	46	Female	East Asia	HBV	M0	T1bN0M0	Yes

Etiology: Etiology of liver cancer; MVI: Microvascular Invasion; TNM Groups: Tumor Node Metastasis Groups.
